# Highly Structured Treatment Programs for Addicted Offenders: Comparing the Effects of the Reasoning & Rehabilitation Program and DBT-F

**DOI:** 10.3389/fpsyt.2020.499241

**Published:** 2020-11-13

**Authors:** Anne Wettermann, Birgit Völlm, Detlef Schläfke

**Affiliations:** Clinic of Forensic Psychiatry, University of Rostock, Rostock, Germany

**Keywords:** forensic psychiatry, addicted offenders, substance misuse, Reasoning and Rehabilitation Program, Dialectical Behavioral Therapy– Forensic, cognitive skills, § 64 StGB

## Abstract

**Background:**

When treating addicted offenders in a forensic psychiatric setting, a primary concern is to decrease antisocial cognitions and behaviors. The cognitive style of offenders is often characterized by impulsiveness, egocentricity, irrational thinking, and rigidity. We examined the relative efficacy of Reasoning and Rehabilitation Program (R&R) and Dialectical Behavioral Therapy– Forensic (DBT-F) on the domains of underlying psychological constructs (e.g., mental flexibility, planning, and problem-solving).

**Materials and Methods:**

The R&R and DBT-F were introduced in a forensic-psychiatric hospital for offenders with substance addictions in Germany. We compared pre- and post-tests to measure the cognitive skills of addicted offenders having undergone R&R (N = 47), DBT-F (N = 34), or Treatment as Usual (TAU; N = 28). Participants’ skills (cognitive flexibility, ability to inhibit cognitive interference, cognitive performance/mental speed, divergent and convergent reasoning/problem solving) were assessed using neuropsychological instruments. Analyses of variance were conducted to investigate whether there were significant improvements within groups and whether these differences were significant between groups. To examine the predictive power of treatment-program on outcomes, and diagnosis of personality disorder, a hierarchical regression model was used.

**Results:**

Both programs were associated with improvements in nearly all of the measured constructs. The only construct on which the R&R and DBT-F groups differed significantly was word fluency, with those receiving R&R improving more than those receiving DBT-F. A regression model showed no predictive power for age, IQ, or diagnosis of personality disorder. Treatment group explained 13.8% of variance in cognitive flexibility but did not predict variance in other outcomes.

**Conclusion:**

Surprisingly, we did not find superiority for one intervention over TAU or differential effects between the two programs. Future research should use larger samples and additional outcomes, including recidivism, to identify possible effects of treatment programs. Additionally, qualitative methods might inform us about these programs are implemented as well as which outcomes may be relevant.

## Introduction

Under § 64 Strafgesetzbuch [StGB (German Criminal Code); (1)], courts can order individuals convicted of an offense to undergo addiction treatment if they suffer from a substance use disorder linked to their offense. Furthermore, “such order is only to be made if there is a sufficiently reasonable prospect that the person can be cured [ … ] by way of placement in an addiction treatment facility or that a relapse into addictive behavior and the commission of serious unlawful acts caused by that proclivity can be prevented for a substantial period of time.” [§64 StGB; (1)]. The maximum length of stay is related to the prison sentence given at the same time, specifically it cannot be longer than two years plus two thirds of this prison sentence but is usually much shorter than that. The average length of stay is two years (2).

Mentally disordered offenders (mostly diagnosed with psychotic disorders, severe intellectual disabilities, or disorders of sexual preference) are treated in forensic-psychiatric hospitals according § 63 StGB on the condition that they have committed an offense in a state of criminal irresponsibility or of diminished responsibility. In addition, the person must represent a danger to the general public due to the risk of committing a serious offense in the future.

In 2013 (latest official figures), there were over 3,600 persons in forensic-psychiatric hospitals detained under § 64 StGB (3). In 2009—the starting point of our project—a survey identified that the §64-population is mostly male (almost 95%) and has an average age of 33.4 years. The patients had on average 8.5 offenses prior to admission, almost 40% were addicted to alcohol or psychotropic medication and 60% to illegal drugs (4). Seventy percent of the population was not diagnosed with a personality disorder (PD), about 13% had an antisocial PD (ASPD), about 4% an emotionally unstable PD, and almost 10% combined PD. The most common index offense was bodily harm (about 32.5%), sex offenses (about 28%), and drug offenses (about 22%). Information on criminal responsibility was not collected at that time.

More recent data from the same longitudinal study indicated that this population is still characterized by male gender (95.2%), an average age of 34.46 years, but that the largest group of index offenses are now drug-offenses (about 33%), followed by bodily harm (26%), and robbery (21%) (5). Individuals had an average of 9.61 previous convictions. Thirty nine percent were diagnosed with polytoxicomania, 19% had an alcohol related disorder, about 13% were addicted to cannabinoids, 10% to opioids, 9% to cocaine, 8% to stimulants, and less than 1% were addicted to sedatives or hypnotics. About 24% of the male forensic inpatients had an ASPD, 9% an emotionally unstable PD, 18% a combined PD, and around 66% had no PD. About 64% of the 2018-population were fully criminally responsible. In sum, the patients have gotten older, have a greater number of previous convictions, are less addicted to alcohol, but increasingly polytoxicomanic and/or addicted to illegal substances. The vast majority is criminally responsible and has no psychiatric co-morbidity.

The recidivism rates of these offenders are up to 50%, 3 years after discharge from inpatient treatment (6–9), considerably higher than those of forensic inpatients with other mental disorders (around 5% after three years in § 63 population). Seifert et al. (10) examined recidivism rates of § 63 offenders for a follow-up period of 16.5 years on average (N = 321). They observed re-offenses in about one third of released forensic patients (35.2%), severe criminal acts (violent crimes or sexual offenses) were committed by 12.8%, and 15.6% were detained in a forensic setting again. The authors observed that the risk for recidivism decreased in patients with schizophrenic disorders but only marginally in those with PD (especially for those who committed sexual offenses). Seifert et al. (10) concluded that it is necessary to identify these “high-risk groups”, provide them with more intense follow up, and evaluate the effectiveness of treatment methods and outcomes after release.

A “high-risk group” in the §64-population are patients with premature termination of treatment because of a low prospect of success (under German law, these patients can be referred back to prison). Recidivism rates for these patients amount to 48% within the first year and 73% after 3 years after discharge from prison (9). The severity of offenses is also much higher compared to regularly discharged patients (9).

Alongside treating the substance use disorder, one of the primary concerns is to decrease criminogenic cognitions and behaviors. Numerous studies have examined the relationship between neuropsychological factors and the onset, development, persistence, and desistance of antisocial behavior (11–15), in which executive functions play a significant role. Executive functions comprise diverse cognitive processes and behavioral capabilities. These functions enable individuals to initiate, plan, regulate, sequence, and achieve complex goal-oriented behavior and thought (16–19). Executive functions are conceptualized as higher-order brain functions (of attention, information organization, forward planning, and self-control) which regulate lower level cognitive processes to performance complex tasks (20, 21).

An overview of the relationship between executive functions and antisocial behavior is presented in the meta-analysis of Ogilvie et al. (22), which demonstrates a strong association. Both individuals with psychopathy and with externalizing traits show distinct cognitive-affective dysfunctions (23–25). Affective and inhibitory deficits can materialize or dissipate in individuals with psychopathy depending on whether affective or inhibitory information is congruent with their goal (26–30). Combined, these studies show that persons with externalizing traits display deficits in executive functions and over-react to emotional information (31–34). They are prone to over-allocate cognitive resources to stimuli in situations that are subjectively motivationally significant. This over-allocation reduces the capacity for other executive functions such as inhibition, shifting, and control (35). A lack of problem-solving skills is also associated with executive functioning difficulties (36).

The relationship between social problem-solving and criminal behavior has been thoroughly examined in the literature (37–41). Poor social problem-solving abilities have been hypothesized to lead to criminal behaviors as maladaptive attempts to solve personal or interpersonal problems (40). There is evidence of a relationship between poor executive functioning and negative treatment outcomes such as increased treatment dropout rates and disruptive behavior during treatment (42). These findings have implications for the treatment of addicted offenders. About 50–70% are discharged without completing treatment (43–47). Most of these studies focus on misdirection of the court. Recent findings indicate that some of these risk factors could be taken into consideration for treatment planning to help reduce premature discharge and recidivism (48). These include executive functions, hyperactivity/impulsivity (49) and aggressiveness/irritability (49, 50).

In the last few decades, in line with the evidence, mentally disordered offenders (incl. addicted offenders) have primarily been treated with cognitive-behavioral therapy interventions (CBT), ranging from psychoeducation to multi-professional and multimodal treatment programs. Barnao and Ward (51) distinguish between treatments targeting mental illness and other psychological issues, interventions based on the principles of the Risk–Need–Responsivity model [RNR; (52, 53)], and strength-based models. All interventions have in common the aim to reduce recidivism. According to Müller et al. (54), Dialectical Behavioral Therapy-Forensic [DBT-F; (55, 56)], Mentalization-Based Therapy [MBT; (57–61)], Schema therapy (62, 63), and Transference Focused Psychotherapy [TFP; (64–66)] are the most commonly applied treatment approaches in forensic psychiatry in Germany. However, evidence for their effectiveness in these settings is largely absent.

DBT is a well-known therapy-approach, which is effective for patients with problems in emotion-regulation. For further description see section “the treatment programs”.

MBT is based on the psychodynamic concept of “mentalization”. Bateman & Fonagy (57) developed this psychotherapeutic approach for the treatment of Borderline-PD. Storebø et al. (67) demonstrated the effectiveness of MBT in BPD in a systematic review, although findings were based on a small number of studies of mainly female patients in non-forensic settings. MBT was more effective in reducing self-harm with a Risk Ratio (RR) of 0.62 [95% CI (0.49, 0.80); 3 trials, 252 participants], suicidality at end of treatment [RR 0.10, 95% CI (0.04, 0.30), 3 trials, 218 participants], and depression [SMD −0.58, 95% CI (−1.22, 0.05), 4 trials, 333 participants], compared with TAU. Findings in relation to interpersonal problems, attrition, and adverse effects were inconsistent. An adapted version of MBT has been developed for patients with ASPD (58) and is currently being examined in a multicenter randomized trial (68).

Schema therapy is an evidence‐based treatment for Borderline PD (69–71) and for Cluster C PDs (72, 73). It is an integrative approach adapting CBT and psychodynamic elements, concepts of attachment theory, humanistic psychology, and other psychological approaches. Bernstein et al. (74) adapted Schema therapy for forensic patients with antisocial, narcissistic, borderline, or paranoid PDs and examined the effectiveness in seven forensic hospitals in the Netherlands (75). Male patients (N = 103) with the aforementioned PDs were randomly allocated to Schema therapy or TAU for 3 years of treatment. Over two-thirds had significant levels of psychopathy; nearly all of them were violent offenders. Results showed that the experimental group had significantly better outcomes than the TAU group on a range of variables [lower risk for recidivism, improved strengths and protective factors, decreased PD symptoms, reduced early maladaptive schemas, and facilitated reintegration into the community; (75)]. Due to these findings, Schema therapy has officially been recognized as the first evidence‐based treatment for forensic patients with PD in the Netherlands (76). Additional indications for forensic populations are also reported by other research teams (77–79). Current research examines whether schema modes are central to the change process (80).

TFP is a manualized, psychoanalytic treatment program, which has evidence for Borderline and other severe PDs (66, 81, 82). Fontao et al. (83) monitored the application of TFP in forensic setting in a pilot study. Therapeutic process was assessed over 18 months. TFP participants (N = 12) showed positive changes in personality dimension scores and global psychopathological indices. Based on the small sample size the generalizability of the study results is reduced.

Beside these therapeutic approaches, there exist a number of other offender rehabilitation programs [e.g., Reasoning & Rehabilitation [R&R; (84)]; Enhanced Thinking Skills [ETS; (85)] and treatment programs on aggression, anger and violence (86–90). ETS appears to improve attitudes regarding aggression and violence in patients with a primary diagnosis of PD (89). Doyle et al. (91) found significant improvements in antisocial attitudes, anger regulation and social problem-solving skills in a prisoner group diagnosed with ASPD compared to TAU. Interventions targeting anger and aggression specifically have yielded inconsistent results (86–88). The long-term effect remains unclear however (90).

### The Treatment Programs

#### The R&R Program

R&R is an evidence-based, manualized cognitive–behavioral program. It is recommended as best practice in the S2-guidelines for the treatment of ASPD of the German Association for Psychiatry, Psychotherapy and Psychosomatics [DGPPN; (92)] and the guidelines of the National Institute for Health and Care Excellence [NICE; (93)].

The R&R Program is a special training for criminal offenders, which targets cognitive skills, enabling them to develop and apply more prosocial behavioral alternatives. In summary, R&R focuses on “modifying the impulsive, egocentric, illogical, and rigid thinking of the offenders and teaching them to stop and think before acting, to consider the consequences of their behavior, to conceptualize alternative ways of responding to interpersonal problems and to consider the impact of their behavior on other people, particularly their victims” [(94), p.31]. R&R was conceived for antisociality-related cognitive problems, not for specific problems such as substance abuse related thinking and behavior. It consists of 36 two-hour sessions, which include role-playing, thinking games, learning exercises, dilemma puzzles, and problem solving (95). The training has nine components: problem solving, social skills, negotiation skills, management of emotions, creative thinking, values enhancement, critical reasoning, skills in review, and cognitive exercises [(84); Institut für forensische Psychiatrie Haina e.V. (IFPH), (96)]. This intervention was originally targeted at medium-to high-risk offenders with an IQ above 70 (because participants have to have adequate verbal skills to understand the content), with a lack of cognitive skills (because their antisocial behavior has to be caused by cognitive deficits) and without issues related to major mental illnesses (95). More recently, the Cognitive Centre of Canada [CCC; (97)] has developed new, adapted, specialized and shorter programs that target the needs of specific groups: R&R2 for Antisocial Adults, R&R2 for Antisocial Youths, R&R2 for individuals with ADHD, R&R2 for Girls and Young Women, R&R2 for those with Mental Health Problems, R&R2 for Families and Support Persons, and R&R2 for Antisocial Drivers (see CCC-website).

The evidence is based on a broad base [for an overview see (98)]. A meta-analysis by Tong & Farrington (99), which included 16 evaluations (involving 26 separate comparisons) from 3 countries (USA, Canada, and UK), showed a significant 14% decrease in recidivism for R&R participants compared to controls. The weighted mean effect size (ES) was 1.16 [95% CI (1.09, 1.27); p < 0.0001], based on rearrests or reconvictions. The period of time at risk varied from 3 to 24 months. R&R groups were less likely to reoffend compared to control-groups (for reconviction/rearrests: 20 OR were greater than 1.0, two were exactly 1.0 and three were less than 1.0). Controls had a 16% increase in recidivism compared to R&R participants, both groups did not differ significantly in revocations, violations, and in return to prison. R&R has been shown to be effective in community [11 trials; ES = 1.27 (p <.017)] and institutional settings [15 trials; ES = 1.16 (p <.0005)], and for both low- and high-risk offenders [8 trials; ES = 1.28 (p <.005) vs. ES = 1.12], judged by recidivism rates. There were also no major differences in reductions in reoffending by R&R participants in all three countries. Furthermore, comparisons between programs delivered to those volunteering for treatment and compulsory programs did not reveal differences (voluntary: OR = 1.17; p <.0001; non-voluntary: OR = 1.20; p <.057).

In 2008, a meta-analysis with a larger pool of 19 evaluations, involving 32 separate comparisons, was published (100). Findings only partially confirmed those reported in 2006. The authors found a weighted mean OR of 1.16 [95% CI (1.04, 1.31); p = .011] in recidivism for controls compared with R&R participants. Program effects for low risk offenders were not statistically significant [10 trials; OR = 1.18 (n.s.)], but they were for high-risk-offenders [10 trials; OR = 1.12 (p = .011)]. Cross-country comparisons revealed some differences: R&R was effective in Canada and the United Kingdom but not in the United States. As before, the program was effective in community [12 trials; ES = 1.22 (p = .023)] and institutional settings [21 trials; ES = 1.15 (p = .064)], whether or not it was given on a voluntary basis.

#### DBT-F

DBT has been shown to be the most effective evidence-based intervention in treating individuals with BPD (101, 102). The adapted DBT-F is a multi-professional and multimodal CBT-based program involving individual and group therapy, skills training, a mindfulness group, and patient meetings without professionals [for detailed information see (103)].

In the S2-guidelines for personality disorders (92) and the NICE-guidelines (104) DBT is recommended as best practice in treating BPD but NICE limited the recommendation to women. In recent decades, DBT has been developed and adapted for individuals with other mental illnesses or other clientele [e.g., for forensic patients, adolescents, individuals with substance use disorders, ADHS, etc.; for an overview see (105)].

The most recent systematic review comparing treatment effects in individuals with BPD comprised 75 randomized controlled trials with 4,507 participants, predominantly females (67). More than 16 different kinds of psychotherapy were included. The most commonly applied psychotherapeutic treatments were DBT and MBT (MBT effects were described above), which were compared with TAU, waiting list, and other treatments. Treatment duration varied from one to 36 months. In sum, authors found beneficial effects on all primary outcomes for BPD-tailored psychotherapy compared with TAU. They observed effects of DBT (compared with TAU) for BPD severity with a standardized mean difference (SMD) of −0.60 [95% CI (−1.05, −0.14); 3 trials, 149 participants], self-harm [SMD −0.28, 95% CI (−0.48, −0.07); 7 trials, 376 participants] and psychosocial functioning [SMD −0.36, 95% CI (−0.69, −0.03); 6 trials, 225 participants]. Secondary outcomes showed mixed findings for anger, affective instability, and chronic feelings of emptiness, impulsivity, attrition, interpersonal problems, and adverse effects. Authors summarized, however, these effects were all based on low-quality evidence and could therefore not be considered robust.

## Rationale

In 2009, we started to implement and evaluate two psychological interventions in our forensic clinic: R&R and DBT-F. Our research project at that time (“The Treatment of antisocial addicted offenders”) was funded by the Ministry for Labor, Social Affairs, Health, and Family of the State of Mecklenburg-Western Pomerania in Germany. The research was designed to add to the What Works literature by looking at a subgroup of criminal offenders detained under § 64 of the German Criminal Code. Preliminary results focusing on the effects of R&R in comparison with TAU were published reporting on changes in cognitive style, impulsiveness, and social cognitions (106–108). These findings indicated that mental flexibility, planning and problem-solving could be improved in the R&R-group compared to controls. Now, we present additional data that includes a DBT arm.

We hypothesized that both R&R and DBT-F would show greater improvements compared to TAU on cognitive skills such as problem-solving and reasoning. We further hypothesized that R&R would show better effects than DBT-F. There have been no direct comparisons of the two approaches as far as we are aware. We hypothesize that R&R would show greater effects on cognitive skills compared to DBT.

## Materials and Methods

### Design

A longitudinal, prospective, quasi- experimental design was used. We compared pre- and post-tests regarding cognitive skills of individuals either having undergone R&R, DBT-F, or TAU.

### Participants

Participants were male inpatients, recruited from a forensic-psychiatric hospital. All participants received treatment for substance addiction according to § 64 StGB. During the time period of the study (2009–2019) more patients were treated in these programs, but due to the voluntary nature of research participation and our inclusion- and exclusion criteria, not all patients could be included in the research.

Our inclusion criteria were: male gender, completed detoxication, completed diagnostic process, and completed pre- and post-measures. We excluded women because of the low number (N = 7) and the associated statistical problems. All study-participants had gave informed their consent.

Our exclusion criteria were: diagnosis of schizophrenia or organic disorder, an IQ of less than 80 (Intelligence Quotient according to the Hamburg-Wechsler-Intelligence test for adults [(HAWIE-R; (109)], Wechsler-Intelligenz-Test für Erwachsene [WIE; (110)] or Wechsler Adult Intelligence Scale [WAIS-IV; (111)]. All mental disorders were diagnosed according the International Classification of Diseases 10 [ICD-10; (112)].

### Treatment Allocation

The study run on three wards: One of the three therapy-wards implemented DBT-F, one R&R, and one ward served as the TAU ward, not having implemented either of these two interventions. Patients were assigned to these therapy wards on the basis of clinical indication: impulsive antisocial inpatients to DBT-F, those with significant antisocial cognitions and behavior to R&R. Those with no such issues were allocated to the TAU ward.

Our clinic offers a wide range of interventions to all patients. Therefore, TAU consisted of weekly psychotherapeutic individual and group therapy sessions for the entire treatment period. In addition, patients took part in psychology led psychoeducational groups (drug and alcohol dependency) and anti-aggression training when appropriate. Treatment was delivered by certified clinical psychologists or advanced trainee psychologists working towards this qualification according to the German therapeutics law (PsychThG). Each inpatient was allocated a primary psychologist as well as two primary nurses. In addition to the psychology run treatment groups there are nurse-lead reflection groups. In addition, a social skills group, led by social workers, is offered. Each inpatient is involved in occupational therapy and sport sessions.

DBT-F was implemented in 2009. According to guidance, it involved individual and group therapy sessions once a week with the psychologist, skills-training, and a mindfulness group. The group skills training involved teaching skills in four domains (mindfulness, distress tolerance, emotional regulation, and interpersonal effectiveness). Nurses leaded the skills training with a frequency of 90 min sessions two times a week. Mindfulness skills were practiced weekly in an extra group, leaded by a psychiatrist. In addition, patients met without professionals formally once a week to manage group activities. The adapted version DBT-F includes a “delict analysis”, which has to worked out in individual therapy sessions together with the psychologist. During a long process, patients shall understand their index delict including underlying processes, risk factors and behaviors, and obvious consequences. The final analysis was presented in the rounds. The DBT treatment (including four skills modules mentioned above) lasted about 12 months.

The R&R program was implemented into routine care in 2009. It involved two sessions of 2 h [recommended by IFPH; (96)] with a group of eight to ten inpatients for about 18 weeks. Two certified trainers (psychologists; certified by IFPH) implemented the manualized sessions. To facilitate transfer into daily life, social workers, nurses, and psychologists were trained on R&R in a two-day workshop, led by the R&R Trainers. In addition, staff are informed as part of routine care about the group topics and possible problems of the individual R&R participants.

### Outcome Measures

We implemented pre- (T1) and post- (T2) measurements immediately before and within two to three months after interventions. T1 and T2 for controls were in line with the time points of the assessment of the R&R participants. We collected pre- and post-data on groups run over a period of ten years. We also collected sociodemographic, clinical, and criminogenic data, namely age, school graduation, professional qualification, IQ, length of stay, previous convictions, index offense, diagnosed substance dependence or harmful use, and diagnosed PD.

To compare the changes in different psychological constructs between groups, a psychometric test battery for executive functions was used, including tests for cognitive flexibility, the ability to inhibit cognitive interference, divergent reasoning, and planning. We selected commonly used [overview in (113)] psychometric instruments for assessing these constructs: the Trail Making Test [TMT-B; (114)], Farbe-Wort-Interferenz-Test [FWIT (Stroop-Test); (115)], Turm von London [Tower of London, TL-D; (116)], and Regensburger Wortflüssigkeits-Test [a word fluency test, RWT; (117)]. Mental speed, as the basis of all intellectual performances, was assessed using the Zahlen-Verbindungs-Test [a number-connection test, ZVT; (118)]. For further information see **Table 1**.

**Table 1:** Assessment information.

Psychological construct	Operationalization	Category
cognitive flexibility	Trail Making Test Part B [TMT-B; (114)]	time of performance in sec.
ability to inhibit cognitive interference	Farb-Wort-Interferenztest [”Stroop“-Test, FWIT; (115)]	T-value of interference
cognitive performance/mental speed	Zahlen-Verbindungs-Test [ZVT; (118)]	ZVT-IQ
divergent reasoning	Regensburger Wortflüssigkeits-Test [RWT; (117)]	Percentile of word fluency
problem solving, convergent reasoning, planning	Tower of London [TL-D; (116)]	Percentile of solved problems

#### TMT-B

The TMT is used for assessing set-switching (defined as the ability to flexibly switch attention between competing task-set representations). In the TMT-B, the participants have to draw lines to connect numbers and letters in a numeric and alphabetic sequence (i.e., 1-A-2-B, etc.) as fast and accurately as possible. The time to completion is typically used as an index for performance (119). A series of studies have validated the TMT-B on healthy and individuals with brain injuries [(120, 121); for an extensive overview see (122)].

#### FWIT

Performing the FWIT, participants are required to read three different tables as fast as possible. The first two tables represent the “congruent condition” in which participants have to read the names of colors, printed in black ink and name different color patches. In the third table color-words are printed in an inconsistent color ink (“incongruent condition”) and participants are required to name the color of the ink instead of reading the word. They therefore have to perform a less automated task while inhibiting the interference arising from a more automated task [Stroop effect; (123)]. We assessed, in line with the literature, the ability to inhibit cognitive interference indexed by time to completion and errors, depicted as T-norms. Numerous studies have found the FWIT to be a reliable assessment tool [e.g., (124); overview in (125, 126)], including in forensic populations (127).

#### TL-D

The Tower of London is one of the most commonly uses measures of planning and problem-solving [e.g., (128–134)]. The test contains a board with three vertical pegs of different heights and three different colored balls. The pegs can hold a maximum of three, two, or one ball. Participants have to convert an initial configuration into a goal configuration by moving the balls among the pegs according to a set of rules (e.g., you can only move one ball at a time, touching one ball counts as a move). The test outcome most commonly used is the number of moves to achieve the goal (unless it is too high in which case it is considered an error). Percentile values are used for analysis.

#### RWT

The RWT is an education-adjusted word fluency test, which has been validated in neurological and psychiatric patient populations, including patients with alcohol dependency; interrater reliability for all subtests is very strong (r = .99), test-retest reliability ranges between r_tt_ = .72 and r_tt_ = .89 (117). The test involves formal lexical and semantic streams. The RWT contains parallel tests, which were used in our study (first form at T1, second form at T2). Four subtests are conducted per measure. Participants have to name as many different words as they can in a period of 2 min per subtest. In the formal lexical subtest, subjects have to name words with a given first letter; in the subtest “formal lexically with shifting”, they are required to name words alternating between two given first letters. In the semantic subtest, participants have to name words fitting to a given category (e.g., food), again there is a shifting-form with two given categories (e.g., clothes or flowers). The number of correct answers is transferred to percentile values.

#### ZVT

The ZVT is a mental speed test with very strong reliability (test-retest reliability between r_tt_ = .84 and r_tt_ = .97; parallel test reliability between r = .95 and r = .98) and a validity of between r = .40 and r = .83 [correlations with various intelligence tests; (135)]. Participants have to draw lines to connect the numbers 1 to 90 in a numeric sequence four times as fast and accurately as possible. The time to completion gives an indication of IQ.

### Statistical Analysis

All analyses were performed with SPSS software Version 24.0 (136). The (at least) ordinal scaled data (data were non-parametric, tested by Shapiro-Wilk) were analyzed by Kruskal-Wallis Test. School education, professional qualification, diagnosis, PD, and index offence were analyzed using chi-square tests. If expected cell frequencies were below five, the Likelihood-quotient was used. Continuous parameters are shown as means and standard deviations, categorical parameters as percentages.

To examine interaction effects between treatment groups, we used a mixed ANOVA-Model. Because the mixed ANOVA is relatively robust regarding breaches of normal distribution, no corrections were made. We tested the homogeneity of covariance by Box’s test (137, 138). As we only had two points of measurement, sphericity was given. Error variances were examined with Levene’s test.

We examined a possible relationship between patient characteristics and the results of the tests with a multiple regression model. Hierarchical regressions enable analyzing possible confounders. Known possible predictors should be entered into the model first in order of their importance for outcomes, new possible predictors can be added (139). We chose, besides treatment-group (Model 2), age at T1, IQ, and personality disorder (Model 1), as possible predictors/confounders. We chose IQ because the IQ, measured by instruments mentioned above, includes—amongst other constructs—reasoning, and education-adjusted factors. Because we examined cognitive performances, age could also have had an effect as cognitive performance, like mental speed or reasoning, etc. decrease with age. Personality structure (BPD and ASPD especially) is sometimes also characterized by typical cognitive dysfunctions (see *Introduction*) depending on severity of PD. We included any diagnosed PD as a possible confounder in the analysis.

All chosen predictors fulfilled criteria for multiple linear regressions (no multicollinearity, etc.).

### Ethical Approvals

The study was approved by the ethics committee of Rostock University Medical Center. All participants gave written informed consent in accordance with the Declaration of Helsinki (140).

## Results

### Sample Characteristics

One hundred and forty-one patients were initially included in the study, 32 were drop-outs (“non-completers”).

#### The Drop-Out-Group

The 32 drop-outs including patients with premature termination of their hospital treatment (N = 6), 17 subjects with uncompleted measures, one patient, who was discharged before post-measurement, 2 patients that were referred back to prison due to court decisions, and 6 unclear drop-out-cases.

Of the six patients who dropped out because of referral back to prison due to low prospect of success, five had started with R&R and one with DBT-F. Four patients were between 23 and 25 years old, one 33, and one was 48 years old. Their IQ ranges from 84 to 102 (missing data: N = 1). Five had completed school, one had a professional qualification. Regarding substance dependence or harmful use, the distribution was equal across the whole sample: two patients had problems with alcohol (addiction or harmful use), two with illegal substances (addiction or harmful use), and two with a combination thereof. Three patients had no PD; the number of previous offenses was also high in this group: only one patient had one previous conviction, the other five had between 6 and 15. Two patients of that group had committed violent offenses (e.g., robbery, assault) as index-delict; one manslaughter; two property offenses; one drug offense. The length of stay (before T1) varied between 5 and 12 months (missing data: N = 1).

#### The Examined Sample

We examined 109 male forensic inpatients. The DBT group comprised 34, the R&R group 47 and the control group 28 males (see **Table 2**). Only those who completed the whole program once were included. Sociodemographic and treatment characteristics are shown in **Table 2**. Most of the participants were around 30 years old. The *average age* was 29.71 years (SD = 7.35). There was no statistically significant difference in age between the groups (**Table 2**). The average IQ across all patients was 94.97 (SD = 10.67) with a range from 80 to 122. IQ did not differ between groups.

**Table 2:** Descriptive information.

Descriptive	Category	TAU (N = 28)	R&R (N = 47)	DBT-F (N = 34)	Total	Ch^2^
Mean age (at T1)	Years (SD)	M = 28.54 (7.38)	M = 29.98 (7.87)	M = 30.29 (6.65)	29.71 (7.35)	χ^2^(2) = 2.21, *p* = .331
IQ	Points (SD)	M = 97.07 (11.98)	M = 94.36 (9.24)^a^	M = 94.03 (11.33)	94.97 (10.67)	χ^2^(2) = 1.30, *p* = .523
School graduation	completed	92%*	75%	62%*	77. %	χ²(2) = 8.53, p = .014
Professional qualification	completed	48%^b^	46%^c^	18%*	37%	χ²(2) = 8.01, p = .018
Length of stay (before T1)	Month (SD)	M = 8.73 (3.90)^d^	M = 9.09 (5.30)^e^	M = 6.79 ( 6.29)*	8.18 (5.55)	χ^2^(2) = 8.08, *p* = .018

*p < .05.

TAU, Treatment as usual; R&R, Reasoning & Rehabilitation Program; DBT, Dialectical Behavioral Therapy-Forensic; M, Mean; SD, Standard Deviation; IQ, Intelligence Quotient according HAWIE- R (109); WIE (110), WAIS-IV - Fourth Edition (111).

^a^n = 44.

^b^n = 25.

^c^n = 46.

^d^n = 15.

^e^n = 43.

The average *lengths of stay (at T1)* of DBT-F-patients was significantly shorter than in the other groups (DBT-F versus R&R p = .014; versus TAU p = .048), but there was no difference between the R&R and the DBT-F group. The number of months at T1 varied from 2 (DBT-F group) to 18 (DBT-F and TAU) months, outliers (38, 26, and 23 months) were removed.

We found no significant differences in *education* and *professional qualification* (see **Table 2**), but a larger proportion of the inpatients of the TAU group had graduated from school than statistically expected, whereas the DBT group comprised fewer than expected. Regarding school dropouts, data are the other way round.

Only 18% of the DBT group and a little less than half of the TAU and R&R participants had a *professional qualification*. This difference was statistically significant.

Offending and diagnostic characteristics are reported in **Table 3**. The majority (55%) of the study participants’ main *offenses* were violent offenses (e.g., robbery, assault), followed by property (13.76%), drug offenses (11.93%), and homicide [11.01% (murder, manslaughter and grievous bodily harm resulting in death)]. Sex offenses were rare. The variable *other offenses* included arson and traffic offenses. Groups did not differ significantly.

**Table 3:** Clinical and Criminogenic Characteristics.

Descriptive	Category	TAU (N = 28)	R&R (N = 47)	DBT-F (N = 34)	Total	Ch^2^
Previous Convictions	Mean of number (SD)	M = 7.64 (4.85)	M = 9.17 (6.00)	M = 10.47 (6.89)	9.18 (6.07)	χ²(2) = 2.78, p = .250
Index offense (frequencies and percentage)	homicide	3 (10.71%)	7 (14.89%)	2 (5.88%)	11.01%	χ²(10) = 8.24, p = .606
	other violent offenses	15 (53.57%)	24 (51.06%)	21 (61.76%)	55.05%	
	property offenses	2 (7.14%)	8 (17.02%)	5 (14.71%)	13.76%	
	drug offenses	5 (17.86%)	4 (8.51%)	4 (11.76%)	11.93%	
	sex offenses	2 (7.14%)	1 (2.13%)	0	2.75%	
	other offenses	1 (3.57%)	3 (6.38%)	2 (5.88%)	5.50%	
Substance dependence or harmful use (frequencies and percentage)	Alcohol	10 (35.71%)	16 (34.04%)	12 (35.29%)	34.86%	χ²(8) = 4.37, p = .822
	Illegal drugs	11 (39.29%)	16 (34.04%)	11 (32.35%)	34.86%	
	Alcohol and illegal drugs	7 (25%)	15 (31.91%)	11 (32.35%)	30.28%	
Personality Disorder or distinctive Personality-Style	no	20 (71.43%)	24 (51.06%)	11 (32.36%)	50.46%	χ²(10) = 20.95, p = .021*
	Cluster A	1 (3.57%)	1 (2.13%)	0	1.83%	
	Cluster B	4 (14.29%)	18 (38.30%)	19 (55.88%)	37.61%	
	Cluster C	0	1 (2.13%)	0	0.92%	
	combined	3 (10.71%)	3 (6.38%)	4 (11.76%)	9.17%	

*A chi-square test was used to compare the variables Personality Disorder (DSM-IV) or distinctive Personality-Style and Treatment group. 61.1% of cells of expected frequencies were below 5, so Likelihood-Quotient was used. Results show a significant between Personality Disorder (DSM-IV) or distinctive Personality-Style and Treatment group, χ²(10) = 16.18, p = .021, φ = 0.39.

TAU, Treatment as usual; R&R, Reasoning & Rehabilitation Program; DBT, Dialectical Behavioral Therapy-Forensic; M, Mean; SD, Standard Deviation.

Disorders due to psychoactive substance use were diagnosed according ICD-10 (112), personality disorders according DSM-IV (141).

In all three groups, the number of *previous offenses* was high. On average, patients in the DBT-group had more than ten (M = 10.47; SD = 6.89) previous convictions, the R&R patients more than nine (M = 9.17; SD = 6.00), and those in the control group greater than seven previous convictions (M = 7.64; SD = 4.85). Previous convictions ranged from 0 to 32. There was no difference between the groups in offending data.

Regarding *substance dependence or harmful use* were no differences between the three groups observed. Approximately one third of each sub-sample was addicted to alcohol, illegal drugs, or a combination thereof (**Table 3**).

Differences in PD *comorbidity* had a non-significant medium effect size (V = 0.27). Half (50.46%) of the patients did not have any PD. The largest group of personality disorders was ASPD and other Cluster B PDs [Diagnostic and Statistical Manual of Mental Disorders; DSM-IV, (141)], followed by a combined PD. More inpatients of the DBT group had a Cluster B PD, and fewer had no PD, than statistically expected. TAU participants frequently had no PD and were less likely to be diagnosed with Cluster B PD. Because of a single inpatient in the R&R group observed data in Cluster C PD was more than expected.

### Comparison of TAU, R&R, and DBT-F Groups on Cognitive Skills

First, we analyzed differences between the three groups in assessed cognitive skills at T1. There were no significant differences in any of the dependent variables (see **Table 4**). At T2, the three groups also did not differ significantly on any of the outcomes assessed. With the exception of ZVT-IQ, the performances (at T1 and T2) were on average within normal ranges in all three groups. In all three groups, ZVT-IQs were below average (at T1 and T2).

**Table 4:** Between-group Differences at T1 and T2: Kruskal-Wallis-Test.

Instrument		Ch^2^	
		at T1	at T2
**TMT-B**: cognitive flexibility		χ²(2) = .09, p = .956^a^	χ²(2) = 3.17, p = .205^e^
**FWIT**: ability to inhibit cognitive interference		χ²(2) = .46, p = .796^b^	χ²(2) = .22, p = .894^f^
**ZVT**: cognitive performance / mental speed		χ²(2) = .51, p = .774^c^	χ²(2) = .01, p = .994^e^
**RWT**: divergent reasoning	formal-lexically	χ²(2) = 2.45, p = .294^d^	χ²(2) = .46, p = .793^g^
	forma-lexically with shifting	χ²(2) = 1.93, p = .382^a^	χ²(2) = .12, p = .940^h^
	semantic	χ²(2) = 3.13, p = .209^a^	χ²(2) = 1.36, p = .506^f^
	semantic with shifting	χ²(2) = .65, p = .721^a^	χ²(2) = .97, p = .614^f^
**TL-D**: problem solving, convergent reasoning, planning		χ²(2) = 1.58, p = .453^c^	χ²(2) = .19, p = .906^h^

TMT, Trail Making Test Part B [TMT-B; (114)]; FWIT, Farb-Wort-Interferenztest [”Stroop“-Test, FWIT; (115)]; ZVT, Zahlen-Verbindungs-Test [ZVT; (118)]; RWT, Regensburger Wortflüssigkeits-Test [RWT; (117)]; TL-D, Tower of London [TL-D; (116)].

^a^n = 106.

^b^n = 108.

^c^n = 104.

^d^n = 107.

^e^n = 97.

^f^n = 98.

^g^n = 99.

^h^n = 100.

#### Within-Group Comparisons

**Table 5** shows the within-group differences between pre- and post-measurement. The TAU group showed significant increases in *cognitive flexibility* with an ES of r = .39; *ability to inhibit cognitive interference* (r = .41); *cognitive performance/mental speed* (r = .33); and *problem-solving/convergent reasoning* (ro = .31).

**Table 5:** Within-group Differences in pre- and post-measurements of TAU, R&R, and DBT-F: Wilcoxon-Test.

Instrument		TAU(n = 21)Z	p	DBT-F(n = 29)Z	p	R&R(n = 45)Z	p
**TMT**: cognitive flexibility		- 2.56	.011*	- 3.71	.000**	- 2.37	.018*
**FWIT**: ability to inhibit cognitive interference		- 2.65	.008**	- 3.08	.002**	- 2.49	.013*
**ZVT**: cognitive performance / mental speed		- 2.04	.041*	- 1.82	.069*^a^	- 2.67	.008**
**RWT**: divergent reasoning/ word fluency	formal-lexically	- .09	.931	- .06	.951	- 1.95	.051*^a^
	forma-lexically with shifting	- 1.17	.242	- 2.37	.018*	- 2.16	.031*
	semantic	- 1.43	.153	- 2.06	.040*	- 2.26	.024*
	semantic with shifting	- .86	.389	- 2.84	.005**	- 2.47	.014*
**TL-D**: problem solving, convergent reasoning, planning		- 2.04	.041*	- 3.14	.002**	- 3.91	.000**

*p < .05.

**p < .01.

TMT, Trail Making Test Part B [TMT-B; (114)]; FWIT, Farb-Wort-Interferenztest [”Stroop“-Test, FWIT; (115)]; ZVT, Zahlen-Verbindungs-Test [ZVT; (118)]; RWT, Regensburger Wortflüssigkeits-Test [RWT; (117)]; TL-D, Tower of London [TL-D; (116)]; TAU, Treatment as usual; R&R, Reasoning & Rehabilitation Program; DBT, Dialectical Behavioral Therapy-Forensic.

^a^p has to halve and compare with alpha, because a one-sided effect (induced increasing by treatment) was expected.

The DBT-F group showed significant increases in *cognitive flexibility* (ES: r = .48); *ability to inhibit cognitive interference* (ES: r = .39); *cognitive performance/mental speed* (ES: r = .23); and *problem-solving/convergent reasoning* (ES: r = .39). Patients in the DBT-F group also demonstrated significant decreases in three of the four subtests of the RWT (*formal lexically with shifting; semantic; semantic with shifting*).

The R&R-group showed significant increases in the following variables: *cognitive flexibility* (ES: r = .25); *ability to inhibit cognitive interference* (ES: r = .26); *cognitive performance/mental speed* (ES: r = .28); *problem-solving/convergent reasoning* (ES: r = .43) and in three of the subtests of the RWT with medium effect sizes: *formal lexical* (ES: r = .21); *formal lexical with shifting* (ES: r = .23) and *semantic* (ES: r = .24). Only in one subtest the T2-results were lower than the T1-outcome (*semantic with shifting*: ES: r = .26).

#### Between-Group-Comparison

There were significant main effects for *cognitive flexibility* (TMT-B), *problem solving/convergent* *reasoning* (TL-D), and *the ability to inhibit cognitive interference* (FWIT), but no statistically significant interaction between these performances and treatment-groups (see **Table 6**). This means average scores were higher post compared to pre-treatment though independent of group membership.

**Table 6:** Results of the mixed ANOVA-Model.

Instrument		main effect	p	interaction effect with group	p
**TMT**: cognitive flexibility		F(1,92) = 24.11	<.001**	F(2,92) = 1.88	.158
**FWIT**: ability to inhibit cognitive interference		F(1,94) = 6.37	.013*	F(2,94) = .44	.648
**ZVT**: cognitive performance/mental speed		F(1,89) = 2.87	.094	F(2,89) = .74	.482
**RWT**: divergent reasoning/ word fluency	formal-lexically	F(1,94) = .05	.822	F(2, 94) = 1.16	.317
	formal-lexically with shifting	F(1,94) = .36	.552	DBT: F(1,29) = 6.84 R&R: F(1,44) = 4.58	.014* .038*
	semantic	F(1,89) = 2.87	.094	DBT: F(1,28) = 4.86	.036*
	semantic with shifting	F(1,93) = 12.06	.001**	F (2, 93) = 1.52	.223
**TL-D**: problem solving, convergent reasoning, planning		F(1,92) = 33.33	<.001**	F(2, 92) = 1.00	.370

*p < .05.

**p < .01.

TMT, Trail Making Test Part B [TMT-B; (114)]; FWIT, Farb-Wort-Interferenztest [”Stroop“-Test, FWIT; (115)]; ZVT, Zahlen-Verbindungs-Test [ZVT; (118)]; RWT, Regensburger Wortflüssigkeits-Test [RWT; (117)]; TL-D, Tower of London [TL-D; (116)]; DBT, Dialectical Behavioral Therapy-Forensic; R & R, Reasoning & Rehabilitation Program.

We did not find any indication for a significant main or interaction effect for *cognitive performance/mental speed* (ZVT) though the within-group-analysis (see below) revealed improvements in all three groups.

The effects of the subtests of RWT (*divergent reasoning*) were heterogeneous. Whereas performance in the *formal lexical subtest* did not change significantly in any of the three groups (no main and no interaction effect), the performance in the *semantic test with shifting* showed a main, but no interaction, effect with the different kinds of interventions. The main effect of the *semantic test with shifting* suggests that all groups demonstrated a decrease in performance.

The only test performances that were dependent on group membership were the RWT subtests *formal lexical with shifting test* and *semantic*. There were statistically significant decreases between T1 and T2 for the DBT-F-participants in *formal lexical with shifting test* and the *semantic subtest*. The scores of the R&R group increased between T1 and T2 (see **Table 6**).

### Multiple Regression

Our results revealed main effects for some test performances, but no statistically significant interaction effect between these performances and treatment groups. We found within-group improvements for all assessed cognitive skills, especially for the R&R group. In order to explore the relationships between the assessed cognitive skills and treatment and/or patient characteristics as predictor variables we used a multiple regression analysis (**Table 7**).

**Table 7:** Results of Multiple Regression.

Instruments		Model I: Age at T1, IQ, PD	p	Model II: Treatment group	p
**TMT**: cognitive flexibility		F(7,78) = 1.56	.161	F(9,78) = 2.39	.020*
**FWIT**: ability to inhibit cognitive interference		F(7,78) = 1.15	.345	F(9,78) = .87	.557
**ZVT**: cognitive performance/mental speed		F(7,74) = .88	.527	F(9,74) = .92	.515
**RWT**: divergent reasoning/ word fluency	formal-lexically	F(7,77) = .42	.889	F(9,77) = .73	.680
			
	formal-lexically with shifting	F(7,77) = .13	.996	F(9,77) = 1.31	.250
	semantic	F(7,75) = 1.17	.333	F(9,75) = 1.52	.161
			
	semantic with shifting	F (7,76) = 1.19	.376	F(9,76) = .92	.513
**TL-D**: problem solving, convergent reasoning, planning		F(7,76) = .46	.862	F(9,76) = .42	.920

*p < .05.

TMT, Trail Making Test Part B [TMT-B; (114)]; FWIT, Farb-Wort-Interferenztest [”Stroop“-Test, FWIT; (115)]; ZVT, Zahlen-Verbindungs-Test [ZVT; (118)]; RWT, Regensburger Wortflüssigkeits-Test [RWT; (117)]; TL-D, Tower of London [TL-D; (116)].

For Model 1, we chose the following patient characteristics: age at T1, IQ, and diagnosed personality disorder. Model 1 did not explain variance of any of the dependent variables (*cognitive flexibility*, *ability to inhibit cognitive interference*, *cognitive performance/mental speed*, *divergent reasoning*, and *problem solving/convergent reasoning*).

Model 2 (treatment group) explained 13.8% of the variance of *cognitive flexibility* (TMT). The R² for the second model was.24 (adjusted R² = .14) for *cognitive flexibility* (TMT), indicative of a medium goodness-of-fit according to Cohen (142). This model did not explain any variance in the other test-results.

## Discussion

The study compared CBT-based treatments, which were developed and validated for criminal offenders. We examined the outcome of two evidence-based programs (R&R and DBT-F) in an addicted offender population in direct comparison with controls (TAU). Using neuropsychological instruments, participants’ cognitive skills were assessed in a pre- and post-measurement design. The measured constructs were cognitive flexibility, ability to inhibit cognitive interference, cognitive performance/mental speed, divergent reasoning, and problem solving/convergent reasoning. Results demonstrated that none of the treatment groups improved significantly more than the others across the measured outcomes. All three groups improved their performances in nearly all of the applied instruments. The only outcome on which patient improvements were distinguishable between the DBT-F and R&R groups was divergent reasoning. Age, IQ, and diagnosed PD did not confound findings.

Our results were unexpected as we hypothesized that both interventions would be more effective than TAU and that R&R participants would improve more compared to the DBT-F group. Our previous research also indicated that mental flexibility, planning and problem-solving improved more in the R&R compared to the control group (106–108).

The most important finding of this study was the absence of a difference between the treatment groups (DBT-F and R&R). All groups, including TAU, differed from baseline values after treatment. There are several important considerations when interpreting this finding. One interpretation could be that all these treatments, which are based on CBT principles (including TAU) worked in our difficult to treat patient group of addicted offenders. Our method of assigning patients to appropriate treatments according to each patient’s clinical profile seemed to work and resulted in improvements. Patients that needed extra help received this within their assigned treatment program and therefore the mean value changes between the groups ends up similar. Our results could indicate that patients were assigned to the appropriate treatment.

Further, we comment on differences between our current findings and previous findings from our own group which identified improvements in cognitive skills in an R&R-group using data up to 2015. Maybe this study reflects the changes in the §64 clientele in the last years. As mentioned more recently this population has gotten older, has had more previous convictions, and has used more varied and multiple substances. It is conceivable that the change in the population led to a less significant treatment effect compared to previous findings.

Our offenders were more similar to a prison population than a population of mentally ill offenders—they were all criminally responsible (at least partly) and functioned at a reasonable level as indicated by the fact that with the exception of one test all tests were within the normal range. Therefore, it is possible that we did not detect changes due to ceiling effects.

Within this group of patients with good cognitive abilities, TAU participants differed from DBT-F patients in that participants from the DBT-F group were older, had a lower IQ, were less educated and qualified, and had more previous convictions. A greater number of patients in that group had a diagnosis of Cluster B PD. Therefore, TAU participants were less severely disordered, had better cognitive abilities and were perhaps better placed to benefit from psychological treatment. As patients were allocated to treatment groups on the basis of clinical need, not randomly, the TAU patients would have been judged clinically to not need additional interventions as would have been demanded by the RNR principle (52, 53). This means on the one hand that the TAU group is expected to benefit from treatment without additional groups such as DBT-F or R&R. On the other hand, one could argue that the DBT-F treatment was effective simply because a more complex patient group still improved with the treatment.

Another possible explanation for the lack of significant differences between the groups could be that all patients in our hospital receive significant therapeutic input, including long-term individual (eclectic) psychotherapy. Therefore, common factors of psychotherapy as the main cause of therapeutic change, such as problem activation, resource activation, coping, motivational clarification, and therapeutic relationship (143–145) are likely to have impacted significantly on the change process. The additional effects of specific interventions might therefore be marginal, but this would not rule out significant treatment effects of such interventions in settings where TAU is less intense. The results highlight the difficulties in evaluating single treatment programs in clinical practice, especially in forensic hospitals. There are many potential confounders when evaluating treatment programs, which could not be controlled [e.g., program characteristics, context effects, evaluation, and participant characteristics; (146)].

We compared two intervention types, which are based more or less on cognitive-behavioral approaches. Our TAU is also oriented, in line with the evidence, towards this. It is possible that we could not detect any differences due to the similarity in theoretical basis and methods used. The results could also indicate that DBT-F and R&R are less suited to treat the special population of addicted offenders. Neither of these two interventions had specific modules for substance misuse.

The findings could also represent the importance of correct program implementation and maintaining fidelity to manualized treatment regimes. Correct R&R implementation is easier to control than DBT-F implementation. Each R&R session is manualized, and the trainers validate each other, even during the session; after each session participants and trainers evaluate it. DBT-F is a multi-professional approach with its own theoretical background and methods. Implementation is expected on the ward as a whole systems approach but we cannot rule out slippage in adherence to DBT principles in clinical practice.

Finally, it is plausible that that the instruments used in the present study did not assess the psychological constructs we attempted to measure, thus reflecting a problem of construct validity.

It is notable that there were significant results of the fourth RWT subtest across the three groups. The subtest *semantically with shifting* assessed the number of words participants can produce over two minutes for given categories. On close inspection of the content of the task, we hypothesized that an effect was shown, which is more associated with creativity, learning strategies, and school success (147, 148). At T1 the inpatients had to produce words according the categories “sports” and “fruits”, at T2 the categories were “clothing” and “flowers”. With the category “flowers”, the male participants scored lower than the pre-measurement. It is possible these results were unrelated to treatment modality. This outcome seems to be more an educational effect, because names of flowers might not be everyday knowledge for this patient group. This effect might have been particularly relevant in the DBT-F group, because these patients were less likely to have achieved educationally (school education and professional qualification) than the other patients.

In contrast to the literature, we did not find distinct executive dysfunctions at baseline, which is normally associated with antisocial behavior [e.g., (22)]. These findings were independent from diagnosed substance abuse or PD, sociodemographic, and criminogenic data. Our results are unexpected in this respect, especially given a lack of problem-solving skills is related to criminal behavior (37–41). R&R was developed to focus—amongst other skills—on these maladaptive problem solution processes. It is possible that our examined population did not benefit from interventions based on these principles. Their deficits and maladaptive resources seem to be different. The evidence of R&R is primarily based on lower recidivism rates after discharge (98–100). This study examined possible changes in executive functions, thus we cannot yet give statements as to long-term effects in our § 64-population. In contrast to R&R, the evidence of DBT is primarily based on reducing symptoms (often in female populations), like BPD severity, self-harm, and psychosocial functioning (67, 149–151). So, research into the efficacy of DBT, particularly DBT-F or DBT-S in male forensic populations (especially in male addicted forensic populations) is still in its infancy.

### Limitations

We examined a relatively small sample of inpatients in one forensic hospital in Germany, so generalizations of our results to the whole population of treated offenders cannot be made. However, our sample was similar to the profile of patients described in Berthold & Riedemann (5), suggesting they were fairly typical of individuals detained under §64 StGB. Another limitation is the allocation to clinical need, so our study was not an RCT. The implementation of DBT-F and R&R was not examined. The investigated measures were purely cognitive measures within a highly structured context, which makes generalization to real world problem-solving difficult, particularly a ceiling effect was suspected. We examined underlying psychological constructs, not “objective criteria” of recidivism and substance-relapse, so we cannot give statements to long-term effects of the treatment-approaches. Outside these points, the results highlight the difficulties of clinical research, particularly the influence of many potential confounders, which could not be controlled.

### Clinical and Research Implications

In sum, the current results indicate that not all of the special group of addicted offenders benefitted from R&R and DBT-F in relation to cognitive skills. Maybe this special clientele would benefit more from DBT-S (DBT for addicted persons), especially the addicted offenders with no personality disorder. It would also be conceivable to combine DBT-F modules with DBT-S modules or extend the duration of treatment for both R&R and DBT with booster-sessions. From our experience, the transfer from theory to practice is particularly difficult for patients, especially in a closed psychiatric setting. This transfer should be more supported by requesting and practicing treated topics and skills in every-day life on wards. It also seems to be very important to pay attention to indication. Therefore, only medium- to high-risk offenders with an IQ above 70, with impairments in cognitive skills and without major mental illnesses (95) should participate in R&R, and only patients with severe impulsivity in DBT-F. Structured and supervised implementation is fundamental to maintain treatment integrity.

Future research should adopt a RCT design to measure differences in outcomes for these groups. However, adopting an RCT design is not easy within forensic inpatient settings given the ethical concerns regarding the randomization of treatments offered under conditions of deprived liberty. Where RCTs are not appropriate, further quasi-experimental or retrospective studies should be conducted. CBT-based programs should be compared with other kinds of evidence-based treatments such as psychodynamically-oriented programs (e.g. MBT group programs or individual therapy), pharmacological interventions, or substance use disorder-specific treatments.

The implementation process of treatment programs should be evaluated. We also suggest the use of different outcome measures to avoid ceiling effects. To verify our findings, it would be important to compare the results with other samples, e.g. a matched sample of imprisoned offenders or individuals with substance abuse problems only. In addition, future research should include the outcomes of criminal recidivism and substance-relapse. We plan to conduct a qualitative study to explore the findings of the present study in more detail. Patients and staff will be asked for their interpretations of the results and the lack of meaningful differences between the treatment groups.

## Conclusion

Overall, our evidence suggests that that there is clinical utility associated with implementing R&R and DBT in forensic treatment settings. R&R seems to be effective in reducing re-offending, DBT in reducing problems with emotion regulation. We did not find evidence attesting to the superiority of one treatment program over another for addicted offenders. All supplied treatments (TAU, R&R, and DBT-F) resulted in improvements. We could derive some clinical and research implications. Additional research is needed to examine the effectiveness of these programs for male addicted offenders. To further investigate the results, we will continue to examine offender treatment, focusing on using different outcome measures without ceiling effects, and explore issues of implementation.

## Data Availability Statement

The datasets generated for this study are available on request to the corresponding author.

## Ethics Statement

The studies involving human participants were reviewed and approved by Ethics committee of Rostock University Medical Center. The patients/participants provided their written informed consent to participate in this study.

## Author Contributions

AW and DS conceived the study and planned data collection and analysis. AW supervised data collection and data entry, analyzed the date, and wrote the first draft of the manuscript. BV supervised the data analysis and the writing process and critically revised the manuscript. All authors contributed to the article and approved the submitted version.

## Funding

Funding was provided by the Ministry for Labor, Social Affairs, Health, and Family of the State of Mecklenburg-Western Pomerania in Germany.

## Conflict of Interest

The authors declare that the research was conducted in the absence of any commercial or financial relationships that could be construed as a potential conflict of interest.
